# Shedding light on negative cultures in osteoarticular infections: leveraging mNGS to unravel risk factors and microbial profiles

**DOI:** 10.3389/fcimb.2024.1457639

**Published:** 2024-11-25

**Authors:** Haiqi Ding, Jiexin Huang, Lan Lin, Yang Chen, Qijin Wang, Wenbo Li, Ying Huang, Xinyu Fang, Wenming Zhang

**Affiliations:** ^1^ Fuzhou University Affiliated Provincial Hospital, School of Medicine, Fuzhou University, Fuzhou, China; ^2^ Department of Orthopaedic Surgery, the First Affiliated Hospital, Fujian Medical University, Fuzhou, China; ^3^ Department of Orthopaedic Surgery, National Regional Medical Center, Binhai Campus of the First Affiliated Hospital, Fujian Medical University, Fuzhou, China; ^4^ Fujian Provincial Institute of Orthopedics, the First Affiliated Hospital, Fujian Medical University, Fuzhou, China; ^5^ Department of Orthopedic Surgery, Nanping First Hospital Affiliated to Fujian Medical University, Nanping, China; ^6^ Department of Orthopedics, Affiliated Mindong Hospital of Fujian Medical University, Ningde, China

**Keywords:** osteoarticular infection, negative microbiological cultures, metagenomic next-generation sequencing, risk factors, pathogen diagnosis

## Abstract

**Background:**

The objective of this study is to utilize metagenomic next-generation sequencing (mNGS) to analyze the risk factors causing negative microbial cultures, comprehensively delineate the microbial profiles neglected by traditional cultures, and optimize the pathogenetic diagnostic procedure accordingly.

**Research design and methods:**

We enrolled 341 patients diagnosed with OI at our center between 2016 and 2022, and gathered data including age, gender, clinical diagnosis, duration of antibiotic use prior to sampling, microbial culture results, and mNGS results for these patients. According to microbial detection results, risk factors for negative microbial culture and mNGS results were investigated through univariate and multivariate analyses, and the microbial profile in cases with negative microbial cultures was summarized in conjunction with mNGS results. Building upon this, we suggest strategies to enhance the positivity rate of microbial cultures based on clinical experience.

**Results:**

Invasive osteoarticular infection (IOI), multi-infections, rare pathogen infections, and prior antibiotic use are risk factors for negative microbial cultures. When the duration of prior antibiotic use is ≥3 days, mNGS demonstrates significantly higher pathogen detection efficiency than microbial culture. Moreover, the risk of negative microbial culture increases by 4.8 times with the exposure to each additional risk factor (OR=4.043, 95%CI [2.835, 5.765], *P*<0.001). Additionally, over one-third of culture-negative OI involve polymicrobial infections or rare pathogens.

**Conclusions:**

Clinicians should tailor microbial culture strategies based on patient conditions. When needed, they can collaborate with mNGS or optimize microbial culture conditions based on mNGS results to enhance the efficiency of pathogen diagnosis.

## 1 Introduction

According to a research group at the 2018 International Consensus Meeting (ICM), the incidence of infection in all subspecialties of orthopedics in North America ranges from 0.1% to 30.0%, and the healthcare cost per patient ranges from $17,000 to $15,000 (Schwarz et al., 2019; Yagupsky and Ceroni, 2023). Osteoarticular infection (OI) pose severe threats, causing excruciating pain, functional impairment, and potentially life-threatening complications, significantly diminishing patients’ quality of life and imposing a substantial burden on healthcare systems (Yagupsky, 2019; Coulin et al., 2021). Accurate and early diagnosis of pathogens is critical for effective treatment of OI, disease management, and the prevention of infection transmission; unclear pathogen diagnosis is a major cause of patient treatment failure because timely treatment is difficult (Goswami et al., 2022).

Currently, the preferred technique for pathogenetic diagnosis in the clinic is microbiological culture. However, the literature suggests that in up to 42% of infections, microbiological culture fails to find the pathogen (Reisener and Perka, 2018). Treatment outcomes are often suboptimal in culture-negative OI, particularly in prosthetic joint infections (PJI), where culture-negative patients face a four-fold higher risk of requiring revision surgery compared to their culture-positive counterparts (Mortazavi et al., 2011). To enhance the detection efficiency of causative pathogens, clinicians are pursuing multifaceted and comprehensive research endeavors. Molecular diagnostic techniques, such as PCR and metagenomic next-generation sequencing (mNGS), have emerged as promising alternatives (Jacovides et al., 2012; Tarabichi et al., 2018). Compared to traditional methods, mNGS offers several advantages, including the ability to detect pathogens that are challenging to culture or exhibit atypical phenotypic characteristics, as well as the capacity to identify multi-infections (Zhang et al., 2019). In the context of OI, mNGS has proven invaluable for detecting culture-negative cases and identifying rare or unusual causative agents (Fang et al., 2021a; Mei et al., 2023).

Microbiological cultures often fail to identify the causative pathogens due to various factors, such as inadequate sample volumes and delayed sample transportation, which markedly reduces the likelihood of isolating the infecting microorganisms (Jain et al., 2015; Sousa et al., 2021). Moreover, the diagnostic performance of microbiological cultures is influenced by the type of OI, with invasive osteoarticular infections (IOI) posing greater challenges for pathogen identification compared to primary osteoarticular infections (POI) (Fang et al., 2022). Invasive orthopedic joint infections (IOI) are typically caused by invasive medical procedures, such as joint puncture, surgical operations (for example, joint replacement surgery, arthroscopy, etc.), or the introduction of pathogens through blood circulation, caused by invasive dental procedures and venous catheters. Primary osteoarticular infections (POI) occur when local tissue infections or inflammations directly extend to bones and joints, generally in the absence of clear external invasive elements (Klosterman et al., 2021). While previous studies have identified various factors influencing microbial culture positivity rates, a comprehensive clinical evaluation of risk factors associated with negative culture results remains lacking.

This study utilized clinical data from a substantial cohort of OI patients treated at our institution to conduct a comprehensive investigation of risk factors associated with negative microbial cultures. Additionally, we performed an in-depth analysis of the microbial characteristics observed in culture-negative cases of OI. Based on these findings, we advocate for an integrated approach to pathogen diagnosis that incorporates metagenomic diagnostics to improve the detection rate of microbial cultures, ultimately aiming to advance early diagnosis and prompt treatment interventions.

## 2 Patients and methods

### 2.1 Patient selection

With the approval of the Ethics Committee of the First Affiliated Hospital of Fujian Medical University (MRCTA, FMU ECFAH[2015]084-2), we enrolled patients who were diagnosed with OI at our facility between 2016 and 2022. Upon admission, they underwent a comprehensive examination, including blood collection for white blood cell count, measurement of erythrocyte sedimentation rate (ESR) and C-reactive protein (CRP) levels. Additionally, for some patients, we evaluated the white blood cell count, white blood cell differential, and leukocyte esterase (LE) in synovial fluid. All patients underwent microbial culture and mNGS. Pathogenetic information of OI patients is obtained from microbial cultures, mNGS, or PCR. Diagnosis is confirmed by at least two senior orthopedic surgeons, two senior infectious disease experts, and one senior microbiologist referencing the diagnostic criteria established at the 2018 International Consensus Meeting (ICM). The inclusion criteria are as follows: (1) Confirmed diagnosis of OI; (2) adequate samples for pathogen testing, including microbiological culture and mNGS; (3) complete medical records; and (4) agreed to participate in this study. The exclusion criteria were as follows: (1) had comorbidities of other infectious diseases, (2) had comorbidities of malignant neoplasms, and (3) had incomplete medical records. All OI patients included in this study were divided into IOI and POI according to the occurrence of infection, among which IOI included periprosthetic joint infection (PJI), implant-related infection (IRI), surgical site infection (SSI), septic arthritis (SA) and other bone and joint infections caused by open injuries or invasive operations. POI included primary septic arthritis (PSA), osteomyelitis (OM), musculoskeletal tuberculosis (MTB), suppurative spondylitis (SS), and so on. In addition, prosthetic joint infection (PJI) refers to infection around the joint prosthesis caused by bacteria, fungi or other microorganisms after prosthetic joint replacement surgery. The diagnosis of PJI is based on the criteria of the Musculoskeletal Infection Society (MSIS). Additionally, prosthetic joint infection (PJI) is an infection of the area surrounding the joint prosthesis following joint replacement surgery, caused by bacteria, fungi, or other microorganisms, and the diagnosis of PJI is based on the Musculoskeletal Infection Society (MSIS) criteria.

### 2.2 Specimen collection and microbial culture

Specimens were transferred to the microbiology laboratory within half an hour after collection, and microbiological cultures were performed by pretreatment according to the sample type. For tissue specimens, an appropriate amount of specimen was digested and ground with 1 ml of trypsin (Qingdao Haibo Biotechnology Co., Ltd., HBPM0153) in an automatic fast grinder (40 Hz, 90 s) until the tissue samples were homogenized. The tissue homogenates were inoculated on blood culture plates and incubated under anaerobic and aerobic conditions. For liquid specimens (e.g., synovial fluid, pus, wound exudate, etc.), specimens were injected into Bactec Plus/F or BactecPeds Plus/F aerobic and anaerobic blood culture flasks (Becton Dickinson, Germany) and incubated in a Bactec 9050 autos tat (Becton-Dickinson, Germany) incubated in a Bactec 9050 automated thermostat (Bectn-Dickinson, Germany) for 14 days. For implants removed intraoperatively, 400 ml of sterile saline was added for sonication (40 Hz, 5 min) to disrupt the biofilm, which was prepared as an ultrasound lysate and then injected into aerobic culture bottles (9239513; Becton-Dickinson, Franklin Lakes, New Jersey, USA) and anaerobic culture bottles (9293496; Becton-Dickinson) for 14 days.

### 2.3 mNGS

Specimen pre-treatment was consistent with that described in microbiological culture and mNGS assay was performed according to the previously described method (Tarabichi et al., 2018; Wang et al., 2020). Briefly nucleic acid extraction was first performed by taking 500μl of liquid or homogenate and extracting total deoxyribonucleic acid (DNA) using the TIANamp Micro DNA kit (DP316, Tiangen, China) according to the manufacturer’s instructions. Subsequently, library construction and sequencing were performed to randomly fragment the DNA into 200-300 bp fragments. dsDNA HS assay kit (Thermo Fisher Scientific, USA) was used to detect the concentration of the DNA libraries; after cyclisation, the libraries were replicated by rolling the ring to produce DNA nanospheres. The prepared DNA nanospheres were loaded onto sequencing chips and sequenced using the BGISEQ-500 platform (UWIC, China). Ultimately, a bioinformatics analysis was performed to exclude low-quality data and sequences shorter than 35bp. Human genomic sequences (Hg19) were eliminated using Burrows-Wheeler alignment, and the residual data were compared against a microbial database to categorize them into viruses, fungi, bacteria, and parasites.

### 2.4 Interpretation of mNGS results

Interpretation of mNGS results was based on previous literature. Genome coverage was defined as the length of detected pathogen sequences divided by the total length of the reference genome (Street et al., 2017; Miao et al., 2018). Relative abundance at the genus level was defined as the proportion of microbial genera in the same broad category (bacteria, fungi, viruses, parasites) among the detected pathogens. Taking into account previous reports in the literature, the thresholds were set as follows: (1) For common background bacteria including Burkholderia spp, Ralstonia spp, Delftia spp, Sphingomonas spp, Streptomyces spp, Sodaria spp, Aspergillus spp, and Albugus spp, they were also identified as pathogens when the relative abundance at the genus level was ≥80%. (2) The pathogenic species was determined by having the greatest coverage rate and standardized number of reads stringently mapped to pathogen at the species level (SDSMRNS) among the pathogenic genera. (3) When standardized, the Mycobacterium TB complex was judged to be the pathogenic bacteria due to the extremely low nucleic acid output; the number of reads stringently mapped to pathogen in genus level (SDSMRNG). In cases with negative microbiological cultures but positive results for mNGS, the following criteria were used to assess the confidence of the mNGS results (Wang et al., 2019; Xie et al., 2021):(1) The use of a third method such as 16S PCR, which showed results that were consistent with those of the mNGS assay. (2) Based on previous studies, pathogens have been reported to cause osteoarticular infections and the reported clinical features are consistent with those of the patients. (3) Targeted therapy is effective as determined by at least three senior clinicians.

### 2.5 Statistical analysis

Continuous variables with normal distribution were expressed as mean ± standard deviation and count data were expressed as numbers (percentages). Differences between the two groups were compared by chi-square test or McNemar test. All statistical analyses were performed using SPSS 26.0; P < 0.05 was considered statistically significant.

## 3 Results

### 3.1 Demographic characteristics

Our overall cohort included 419 patients with bone and joint infections. Of these patients, 75 were not screened via mNGS, and 3 were excluded due to incomplete follow-up data.Of the 341 enrolled patients, 272 had positive culturing results, while 69 had negative culturing results. There were no significant differences in age, BMI, a CCI, or sex between the two groups. Microbial culture-positive patients included 150 (55.15%) IOI patients, including 123 (45.22%) with PJI. There were 38 (13.97%) patients with multi-infections and 31 (11.40%) patients with infections of rare bacteria identified by mNGS. The duration of antibiotic use before sampling was 2.5 ± 3.6 days. The microculture-negative patients included 51 (73.91%) IOI patients and 42 (60.87%) PJI patients. There were 28 (40.58%) patients with multi-infections and 27 (39.13%) patients with infections caused by rare pathogens identified by mNGS. The duration of antibiotic use before sampling was 9.2 ± 6.0 days. The diagnosis of IOI, PJI, multi-infections, and rare pathogens infections and the duration of antibiotic use before sampling are important factors that may affect the results of microbial culture (**Table 1**).

**Table 1:** Characteristics of patients.

Characteristics	CP (n =272)	CN (n=69)	*P Value*
Age (years)	58.5 ± 16.7	62.1 ± 14.1	0.099
BMI (kg/m2)	24.97 ± 2.73	24.45 ± 2.14	0.092
aCCI	1.89 ± 1.45	2.16 ± 1.36	0.170
Gender (M/F)	122/150	34/35	0.510
Diagnosed with IOI or POI	150/122	51/18	0.005
Diagnosed with PJI, n (%)	123 (45.22%)	42 (60.87%)	0.020
Multi-infection detected by mNGS, n (%)	38 (13.97%)	28 (40.58%)	< 0.0001
Rare pathogen detected by mNGS, n (%)	31 (11.40%)	27 (39.13%)	< 0.0001
Prior use of antibiotics (days)	2.5 ± 3.6	9.2 ± 6.0	< 0.0001

CP, culture positive; CN, culture negative; BMI, body mass index; aCCI, age-adjusted Charlson Comorbidity Index; M, Male; F, Female; IOI, invasive osteoarticular infection; POI, primary osteoarticular infection; PJI, prosthetic joint infection.

### 3.2 Risk factors for negative microbial culture

**Figure 1:**
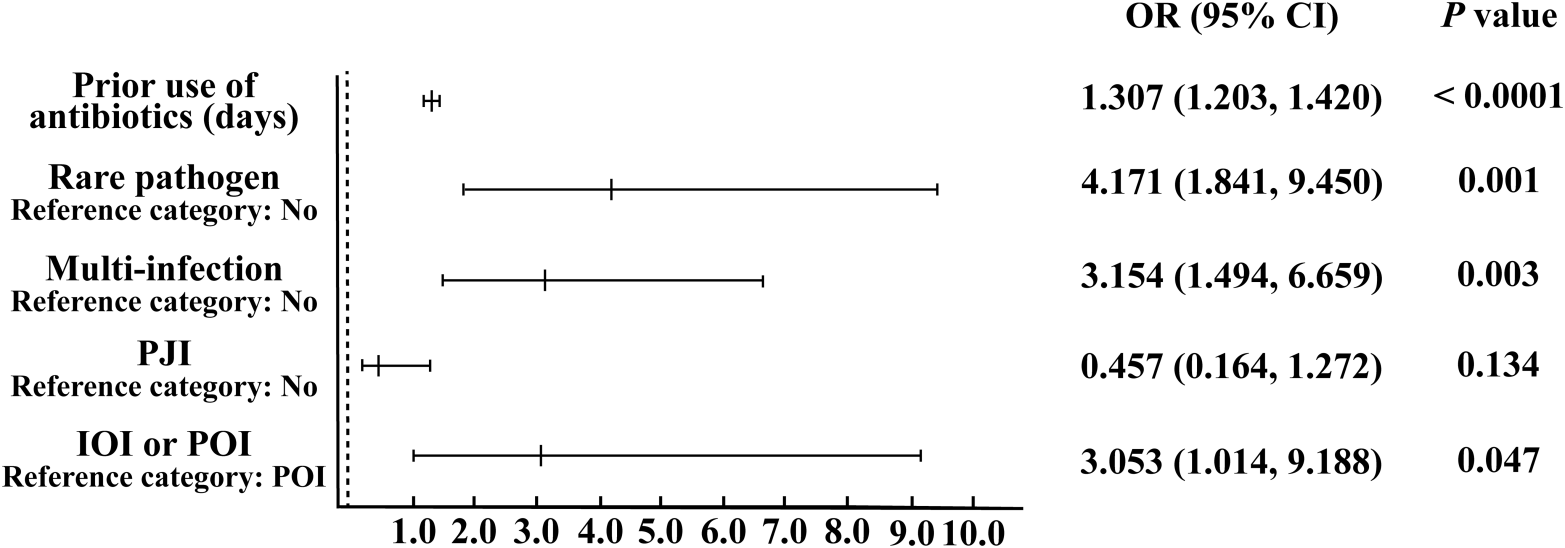
Binary logistic regression analysis of risk factors leading to negative microbial culture results.

According to the analysis results presented in **Table 1**, we utilized IOI, PJI, multi-infections, rare pathogens infections, and the use of antibiotics before sampling to construct a multifactor logistic regression model. The results showed (**Figure 1**) that for each additional day of antibiotic use before sampling, the probability of negative microbial culture significantly increased by 30.7% (OR=1.307, 95%CI [1.203, 1.420], P< 0.0001). The risk of culture-negative infection in patients with rare pathogens infection was 4.171 times greater than that in patients with nonrare pathogens infection, and the difference was significant (OR= 4.171, 95%CI [1.841, 9.450], P= 0.001). The risk of culture-negative infection in patients with multi-infections was 3.154 times greater than that in patients without multi-infections, and the difference was significant (OR= 3.154, 95%CI [1.494, 6.659], P= 0.003). The risk of culture-negative infection in IOI patients was 3.053 times greater than that in POI patients (OR= 3.053, 95%CI [1.014, 9.188], P= 0.047). There was no significant increase in the risk of negative microbial culture results in PJI patients compared with non-PJI patients (OR= 0.457, 95%CI [0.164, 1.272], P= 0.134).

### 3.3 The advantages of mNGS in detecting pathogen information in patients with OI

**Figure 2:**
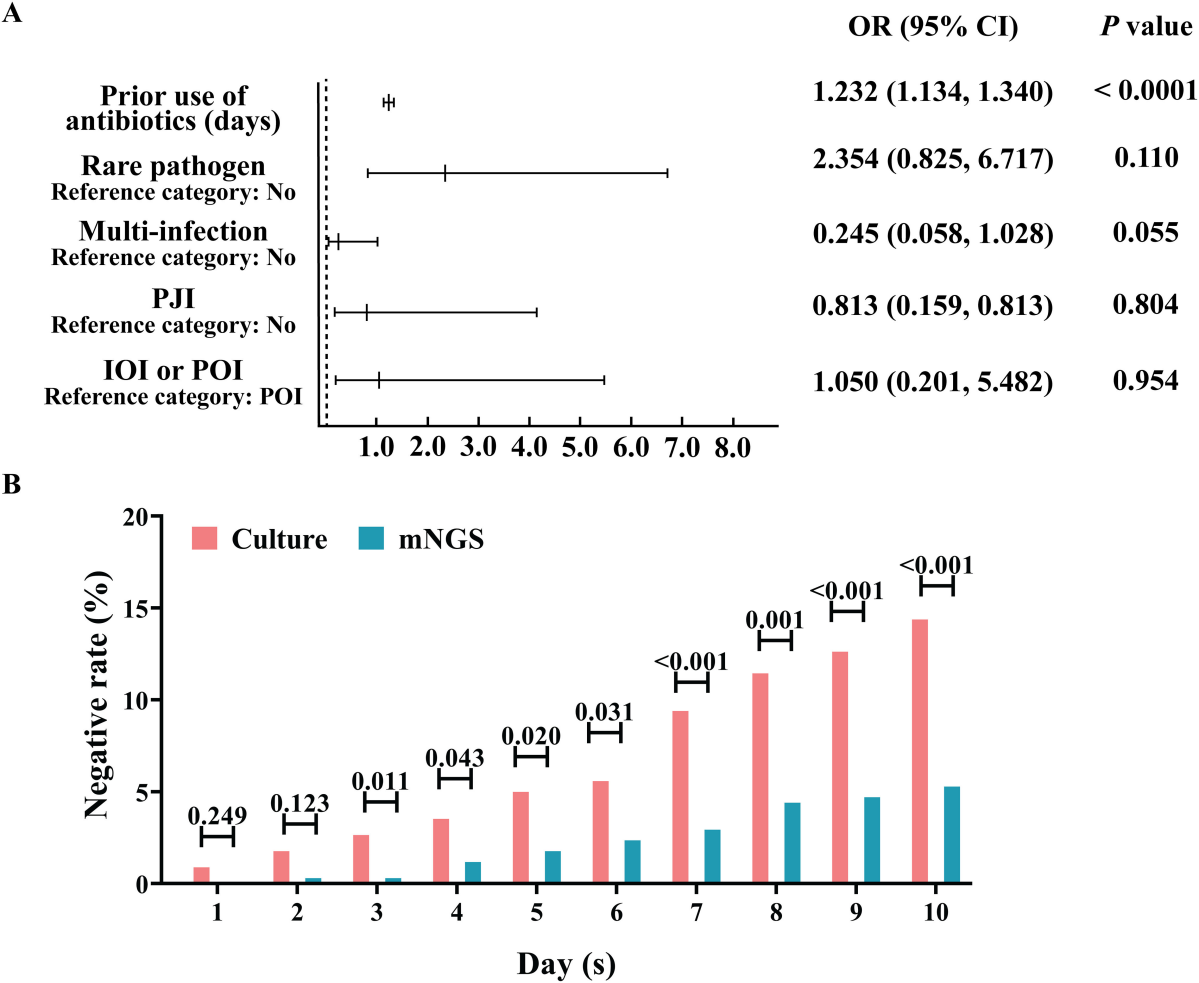
Advantage analysis of the aetiological diagnosis of mNGS. **(A)** Analysis of risk factors for negative mNGS detection. **(B)** Effect of the duration of antibiotic treatment before sampling on the percentage of negative microbial culture results and the mNGS aetiology results.

The percentages of positive microbial culture and mNGS results were calculated (**Table 2**). As shown in **Table 2**, approximately 79.77% (272/341) of the microbial cultures were positive, and 92.67% (316/341) of the mNGS samples were positive; the percentage of positive mNGS samples was significantly greater than the percentage of positive culture samples (P<0.0001). With mNGS results as the dependent variable, OI type, PJI, multi-infections, and the use of antibiotics before sampling were analysed to construct a multivariate logistic regression model. We found that (**Figure 2A**) IOI, PJI, multi-infections, and rare pathogens infections were not risk factors for mNGS negativity, but the probability of a negative microbial culture significantly increased by 23.2% for each additional day of antibiotic use before sampling (OR=1.232, 95%CI [1.134, 1.340], P<0.0001). In addition, stratification was conducted according to the duration of antibiotic use before sampling. The analysis results indicate that when antibiotics are used for ≥3 days prior to sampling, the rate of negative results in mNGS is significantly lower than that in microbiological culture (**Figure 2B**). Further analysis revealed that for risk factors including IOI, multi-infections, rare pathogens infections, and prior antibiotic use, the risk of negative results in microbiological culture increased by 4.8 times with exposure to each additional risk factor (OR=4.043, 95% CI [2.835, 5.765], P<0.001), while the risk of negative results in mNGS increased by 1.849 times with exposure to each additional risk factor (OR=1.849, 95% CI [1.257, 2.719], P<0.001).

**Table 2:** Pathogenic detection rate of mNGS and microbial culture in IOI and POI.

	Positive	Negative	*P Value*
			<0.0001
Microbial culture	272	69	/
mNGS	316	25	/

IOI, invasive osteoarticular infection; POI, primary osteoarticular infection.

### 3.4 Microbial profiles of culture-negative OI detected by mNGS

**Figure 3:**
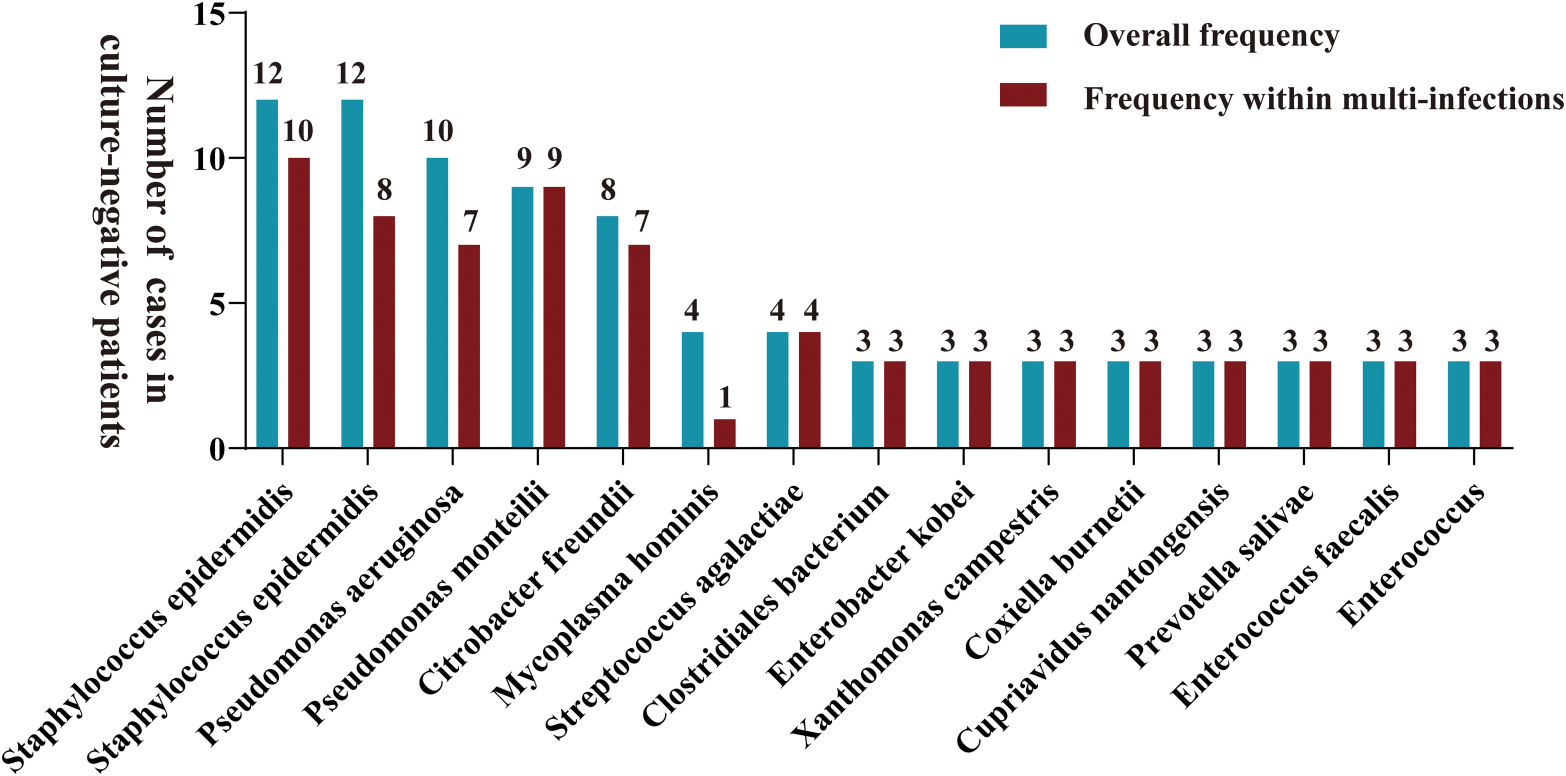
A bar graph is presented for samples with negative microbial cultures, illustrating the overall distribution frequency of diverse microorganisms identified by mNGS (green bars) and their frequency in polyinfections (red bars). Only pathogens that were reported at least three times in the study area were included in mapping.

**Figure 4:**
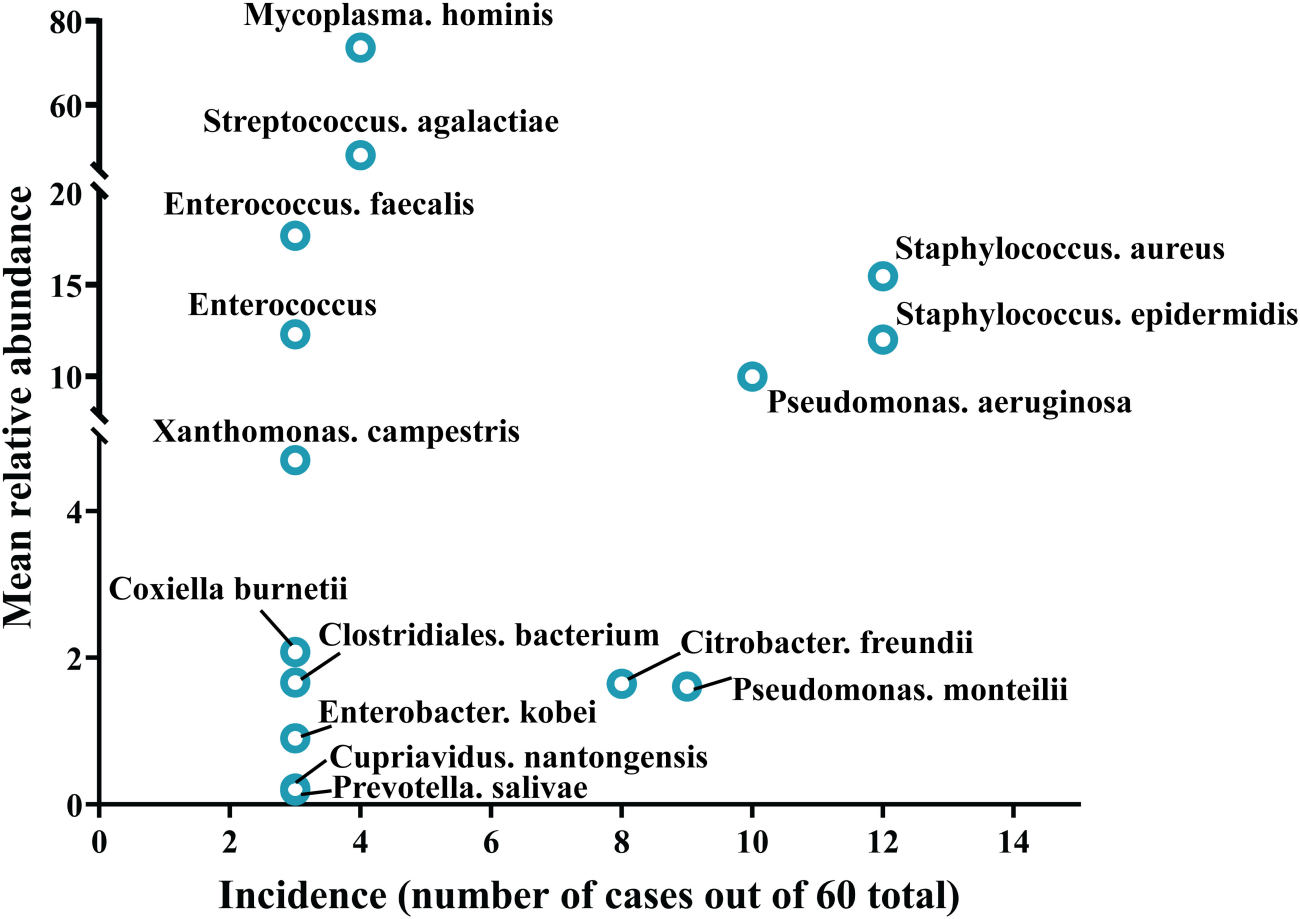
Trends in pathogen incidence and prevalence, as summarized based on the relationship between incidence and study-wide mean relative abundances.

Samples from infection sites of OI patients with negative microbial cultures were subjected to mNGS detection, where 38.71% were ultrasonic dissolution fluid samples, followed by synovial fluid (35.48%) and fresh tissue (25.81%) samples. From the entire cohort, 114 different pathogens were detected by mNGS, 45 of which were gram-positive organisms, 56 were gram-negative organisms, 10 were fungi, 2 were mycoplasmas, and 1 was rickettsia (**Supplementary Table S1**). Among these, 13.15% of the pathogenic bacteria (15 out of 114 species) were present in at least 3 patients (approximately 5%). When ranked according to the incidence of culture-negative OI cases, the most common strains were *Staphylococcus aureus*, *Staphylococcus epidermidis*, *Pseudomonas aeruginosa*, *Pseudomonas monteri*, *Limonobacter fraudiensis*, and *Mycoplasma hominis* (**Figure 3**). mNGS revealed that 46.67% (28 patients) of the culture-negative OI patients had multiple pathogenic microorganisms. The median number of pathogens identified in each multiple infection was 5, and the mean and standard deviation were 6.8 ± 5.1 (**Figure 3**). In addition, of the 28 multi-microbial patients, 82.14% had multiple species defined as common (based on a 5% incidence threshold); in these cases, the median and average were 3 and 3.4 ± 2.7 common species, respectively. In the case of multi-infections, the relative abundance of a bacterium was used as an indicator of the dominant microorganism. In our sample set, *Mycoplasma hominis*, *Streptococcus agalactis*, *Enterococcus faecalis*, and *Staphylococcus aureus* were the most common among the multiple microbial infections, while *Staphylococcus aureus*, *Staphylococcus epidermidis*, and *Pseudomonas aeruginosa* had the highest incidence (**Figure 4**).

### 3.5 Representative culture-negative cases diagnosed by mNGS

Representative culture-negative patients that were accurately diagnosed utilizing the mNGS method are listed in **Supplementary Table S2**. Patients 1–5 had PJI, and no pathogenic bacteria were detected via microbial culture. The pathogens detected by mNGS were *Candidatropicalis*, *Coxiella burnetii*, *Fusobacterium nucleatum*, *Mycoplasmahominis*, and *Finegoldiamagna*, all of which were cultured fussy bacteria. Patient 6 presented with sudden knee swelling, pain, and an increased skin temperature. PSA was suspected, but preoperative synovial microbiological cultures were negative. mNGS suggested that the pathogen may be *Parvimonas micra (P. micra)*, an anaerobic bacterium that requires specific culture conditions and is difficult to isolate through conventional culture methods. To confirm the results, 16S PCR was used to identify pathogenic bacteria, and the results were consistent with the mNGS results, indicating that P. micra was a true pathogen rather than a background bacterium. Preoperative synovial microbiological cultures in patients 7-9 were negative, but mNGS suggested *Mycobacterium abscess*, *Mycobacterium tuberculosis*, and *Mycobacterium columbiae*, respectively. After treatment with targeted antibiotics, the infections were well controlled. The pathogens in patients 10–12 were *Mycobacterium abscess*, *Mycobacterium tuberculosis*, and *Mycobacterium columbiae*, respectively. Patients 10 and 11 were treated with antibiotics within 2 weeks, resulting in low bacterial viability, a low bacterial load, and negative microbial culture results. Patient 13, had chronic PJI; the sample was a joint prosthesis, and the reason for the negative culture may be related to the immune escape mechanism of *Staphylococcus aureus*. mNGS suggested that the pathogen was *Pseudomonas aeruginosa*, although this bacterium has no special requirements for microbial culture and is generally easy to isolate.

## 4 Discussion

Targeted antibiotic therapy is a crucial strategy for treating OI, highlighting the importance of early etiological diagnosis. Despite the significant contribution of microbial cultures to OI treatment by clinicians over the years, there is still room for improvement in their sensitivity and accuracy in pathogen detection. To explore optimization of microbial culture systems, we collected microbial culture data and mNGS detection data from 341 OI patients at our medical center. Based on the mNGS detection results, we analyzed risk factors and microbial profiles for culture-negative specimens, we found that IOI, multi-infections, rare pathogens and prior antibiotic use (≥3 days) were key risk factors for negative microbial culture. In addition, PJI patients should also be given sufficient attention due to the particularity of their infection environment.As reported in previous studies, the use of antibiotics prior to sample acquisition was one of the significant risk factors associated with negative microbial cultures (Berbari et al., 2007; Kalbian et al., 2020). Our results showed that mNGS is more tolerant to pre-sampled antibiotic use than microbiological culture, and mNGS pathogen detection significantly reduces the percentage of negative results at ≥3 days of pre-sampled antibiotic use. Given the high sensitivity of mNGS for the aetiological detection of bone and joint infections, the use of prophylactic antibiotics when OI is diagnosed or suspected and when the pathogen has not yet been identified depends on the clinician’s judgement based on serological examination of the CRP, RSR, and PCT and clinical manifestations such as local redness, swelling, and pain. Especially for patients with PJI, the International Medicine (ICM) consensus in 2018 indicated that in the case of a diagnosis or suspected PJI, there is no need to force the treatment of unknown pathogens, thus antibiotics can be withheld (Schwarz et al., 2019).

Multi-infection may occur in a proportion of cases, and these factors may lead to uncertainty in the diagnosis of the aetiological agent (Hoffman et al., 2006; Wolcott et al., 2013). In the present study, mNGS revealed that 34/69 patients had mixed infections. In fact, in an infected ecosystem at an orthopaedic surgical site, when the absolute amount of microbial space and nutrients is insufficient, two or more microbial populations compete for the same resources, and the dominant flora may eliminate other microbial species in the same environment or affect the growth rate of microorganisms under symbiosis (Hoffman et al., 2006; Wolcott et al., 2013; Xie et al., 2020). This competitive relationship leads to fewer microbes and, as the number of microbial species increases, the risk of negative culture results increases. Due to the complex environment at the time of injury, the probability of multi-infections is significantly greater in IOI patients than in POI patients. In the present study, we revealed that disease type significantly affects the risk of negative microbial cultures.

In addition, another important reason for negative microbial culture results is infection by rare pathogens (Signat et al., 2011; Chenouard et al., 2019; Anagnostakos et al., 2021; Huang et al., 2022). Among these atypical pathogens, *Mycobacterium tuberculosis* and *Mycobacterium abscessus* requires a special growth medium; otherwise, it is difficult to culture the results (Lasso and Pérez, 2009; Watanabe et al., 2023). For *Mycoplasma*, *Fungi*, and *Propionibacterium acnes* (*P. CNES*), the culture conditions are strict; thus, it can take 21 days to isolate the pathogen (Zappe et al., 2007; Rienmüller and Borens, 2016; Singh et al., 2023). In the case of these atypical pathogens, molecular diagnostic techniques such as polymerase chain reaction or mNGS are often needed to confirm the diagnosis (Huang et al., 2022; Suárez-Cuervo et al., 2022). It is worth noting that IOI encompass a diverse array of causative agents, ranging from common bacterial pathogens to rare etiologies like *Mycobacteria*, *Fungi*, and *Rickettsia*, with a subset of cases involving multi-infections (Thoendel et al., 2017; Huang et al., 2019; Krauth et al., 2021).

Further, patients with implant-associated infection in clinical practice often develop chronic infection, and the pathogenic bacteria can form biofilms on the surface of implants, reducing the number of pathogenic bacteria in tissues and resulting in negative bacterial culture, which is common in patients with chronic PJI (Gbejuade et al., 2014). In recent years, our team showed that *Staphylococcus aureus* can undergo small colony mutations in chronic infections, resulting in low toxicity, low metabolism, and difficulty culturing (Cai et al., 2023). In the above circumstances, although pathogens are difficult to detect, they still possess pathogenicity, and these pathogens exist in a state similar to viable but non-culturable (VBNC) (Xu et al., 1982; Murugan and Vasudevan, 2018; İzgördü et al., 2022). In the VBNC state, microbes cannot be detected by traditional laboratory methods, but they can be revived under the right conditions. Moreover, invading pathogens can form intracellular infections that evade the host’s immune defenses (de Mesy Bentley et al., 2017; Krauss et al., 2019). During chronic infection, *Staphylococcus aureus* can colonize bone cells or bone lacunae; thus, the bacteria can colonize the host for a long time and the number of bacteria outside the cell can be reduced, resulting in negative culture results.

**Figure 5:**
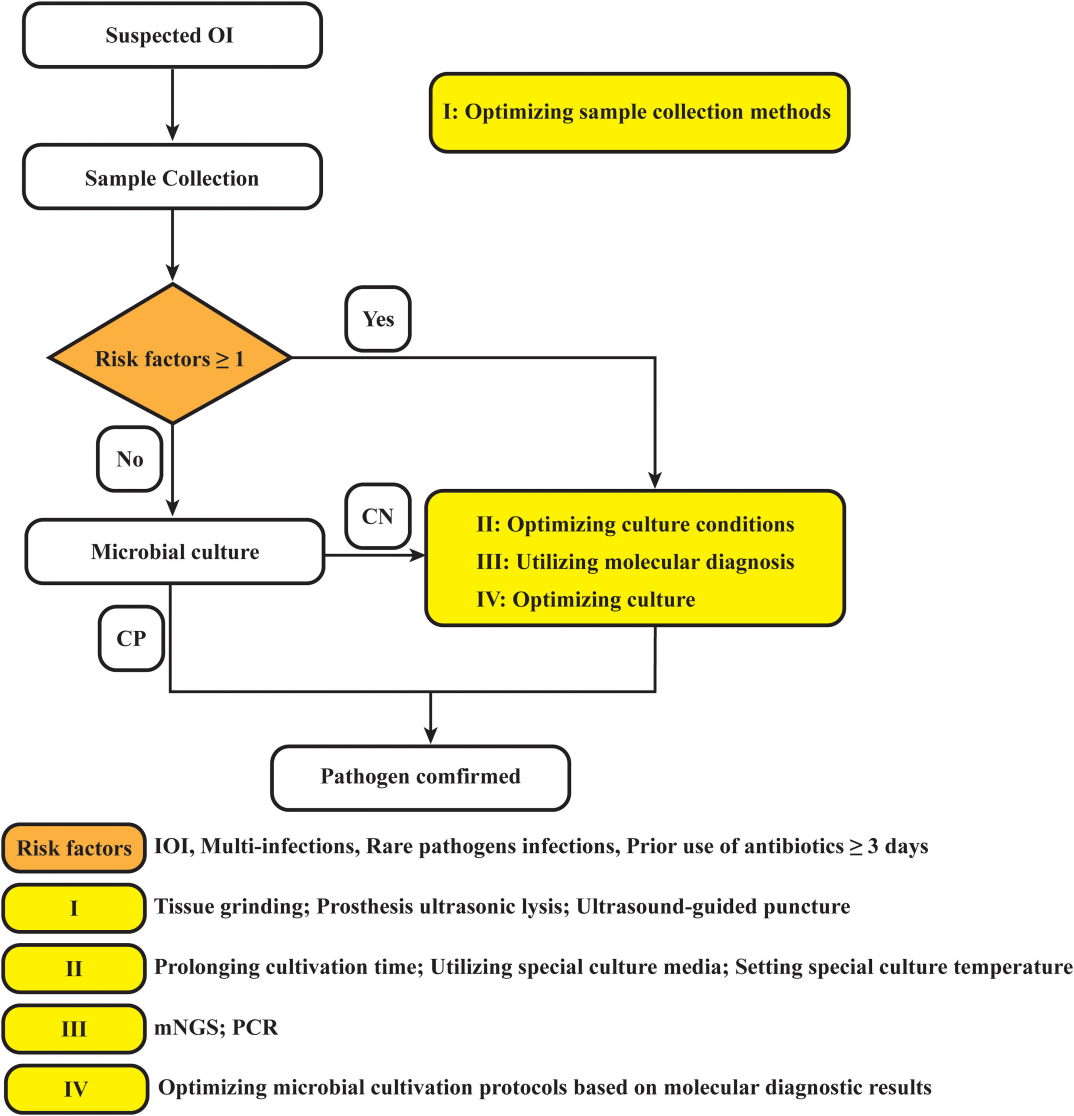
Diagram of the optimized strategy for pathogen detection in osteoarticular infections. OI, osteoarticular infection; CN, culture negative; CP, culture positive; PJI, periprosthetic joint infection; mNGS, metagenomic next-generation sequencing; PCR, polymerase chain reaction.

In summary, based on this study, we propose providing recommendations for optimizing the clinical microbiological testing workflow accordingly (**Figure 5**). In fact, we have gradually applied the above strategies to clinical applications, and early studies by our team have shown that tissue grinding can significantly improve the positive rate of microbial culture (Fang et al., 2021b). Additionally, the work of Bonnet M et al. has demonstrated that modifying microbial culture conditions—such as altering the culture medium, extending incubation periods, and adjusting anaerobic settings—can enhance the positivity rate of microbial cultures (Bonnet et al., 2019). Furthermore, the advantage of mNGS in detecting multiple infections and rare pathogens has been confirmed (Li et al., 2018; Wang et al., 2019; Xiao et al., 2019; Xie et al., 2023; Qian et al., 2024). Although mNGS testing has many advantages over traditional pathogen detection methods, it cannot completely replace traditional methods. Exploring the right timing for using mNGS, expanding potential application scenarios, and combining it with microbial culture plans can optimize the detection of pathogenic microorganisms, thereby better serving patients and physicians.

## 5 Conclusions

In this work, based on the results of mNGS, we identified IOI diagnosis as a significant risk factor for negative microbiological cultures and further identified significant correlations among IOI diagnosis, multi-infections, and PJI diagnosis. Compared to microbiological culture methods, mNGS is more effective at excluding factors such as the diagnosis of IOI, multi-infections, PJI, and the presence of rare organisms, thus significantly improving the detection of pathogenic microorganisms. However, pre-sampling antibiotic use is an important risk factor for negative microbiological culture and mNGS results. Based on the above findings, we further developed a process for pathogenic microbiological diagnosis in the clinic; we believe that this process can provide clinicians with ideas to accelerate the early pathogenic diagnosis of infected patients.

## Data Availability

The data presented in the study are deposited in the China National GeneBank Database (CNGBdb) repository, accession number CNP0001047 (http://db.cngb.org/cnsa/).

## References

[B1] Anagnostakos K. Grzega C. Sahan I. Geipel U. Becker S. L. (2021). Occurrence of rare pathogens at the site of periprosthetic hip and knee joint infections: A retrospective, single-center study. Antibiotics (Basel). 10, 882. doi: 10.3390/antibiotics10070882 34356802 PMC8300814

[B2] Berbari E. F. Marculescu C. Sia I. Lahr B. D. Hanssen A. D. Steckelberg J. M.. (2007). Culture-negative prosthetic joint infection. Clin. Infect. Dis. 45, 1113–1119. doi: 10.1086/522184 17918072

[B3] Bonnet M. Lagier J. C. Raoult D. Khelaifia S. (2019). Bacterial culture through selective and non-selective conditions: the evolution of culture media in clinical microbiology. New Microbes New Infect. 34, 100622. doi: 10.1016/j.nmni.2019.100622 31956419 PMC6961714

[B4] Cai Y. Chen X. Huang C. Chen Y. Zhang C. Huang Z.. (2023). Alteration of m6A-tagged RNA profiles in bone originated from periprosthetic joint infection. JCM. 12, 2863. doi: 10.3390/jcm12082863 37109200 PMC10146075

[B5] Chenouard R. Hoppé E. Lemarié C. Talha A. Ducellier F. Ferchaud F.. (2019). A rare case of Prosthetic Joint Infection associated with Coxiella burnetii. Int. J. Infect. Dis. 87, 166–169. doi: 10.1016/j.ijid.2019.07.028 31374343

[B6] Coulin B. Demarco G. Spyropoulou V. Juchler C. Vendeuvre T. Habre C.. (2021). Osteoarticular infection in children. Bone Jt J. 103-B, 578–583. doi: 10.1302/0301-620X.103B3.BJJ-2020-0936.R2 33641416

[B7] de Mesy Bentley K. L. Trombetta R. Nishitani K. Bello-Irizarry S. N. Ninomiya M. Zhang L.. (2017). Evidence ofStaphylococcus aureusDeformation, proliferation, and migration in canaliculi of live cortical bone in murine models of osteomyelitis. J. Bone Miner Res. 32, 985–990. doi: 10.1002/jbmr.3055 27933662 PMC5413415

[B8] Fang X. Cai Y. Chen X. Huang C. Lin Y. Huang Z.. (2022). The role of metagenomic next-generation sequencing in the pathogen detection of invasive osteoarticular infection. Int. J. Infect. Dis. 122, 996–1001. doi: 10.1016/j.ijid.2022.07.061 35908720

[B9] Fang X. Cai Y. Mei J. Huang Z. Zhang C. Yang B.. (2021a). Optimizing culture methods according to preoperative mNGS results can improve joint infection diagnosis. Bone Jt J. 103-B, 39–45. doi: 10.1302/0301-620X.103B1.BJJ-2020-0771.R2 33380187

[B10] Fang X. Zhang L. Cai Y. Huang Z. Li W. Zhang C.. (2021b). prior pretreatment methods on microbial culture results in the diagnosis of periprosthetic joint infection. Bone Joint Res. 10, 96–104. doi: 10.1302/2046-3758.102.BJR-2020-0104.R3 33517765 PMC7937541

[B11] Gbejuade H. O. Lovering A. M. Webb J. C. (2014). The role of microbial biofilms in prosthetic joint infections. Acta Orthop. 86, 147–158. doi: 10.3109/17453674.2014.966290 25238433 PMC4404764

[B12] Goswami K. Clarkson S. Phillips C. D. Dennis D. A. Klatt B. A. O’Malley M. J.. (2022). An enhanced understanding of culture-negative periprosthetic joint infection with next-generation sequencing. J. Bone Joint Surgery. 104, 1523–1529. doi: 10.2106/JBJS.21.01061 35726882

[B13] Hoffman L. R. Deziel E. D’Argenio D. A. Lepine F. Emerson J. McNamara S.. (2006). Selection for Staphylococcus aureus small-colony variants due to growth in the presence of Pseudomonas aeruginosa. Proc. Natl. Acad. Sci. U.S.A. 103, 19890–19895. doi: 10.1073/pnas.0606756104 17172450 PMC1750898

[B14] Huang C. Ding H. Lin Y. Zhang Z. Fang X. Chen Y.. (2022). Diagnosis of *Coxiella burnetii* Prosthetic Joint Infection Using mNGS and ptNGS: A Case Report and Literature Review. Orthop Surg. 15, 371–376. doi: 10.1111/os.13600 36377682 PMC9837287

[B15] Huang Z. Zhang C. Fang X. Li W. Zhang C. Zhang W.. (2019). Identification of musculoskeletal infection with non-tuberculous mycobacterium using metagenomic sequencing. J. Infect. 78, 158–169. doi: 10.1016/j.jinf.2018.10.002 30312646

[B16] İzgördü Ö.K. Darcan C. Kariptaş E. (2022). Overview of VBNC, a survival strategy for microorganisms. 3 Biotech. 12. doi: 10.1007/s13205-022-03371-4 PMC9526772 36276476

[B17] Jacovides C. L. Kreft R. Adeli B. Hozack B. Ehrlich G. D. Parvizi J. (2012). Successful identification of pathogens by polymerase chain reaction (PCR)-based electron spray ionization time-of-flight mass spectrometry (ESI-TOF-MS) in culture-negative periprosthetic joint infection. J. Bone Joint Surgery. 94, 2247–2254. doi: 10.2106/JBJS.L.00210 23318615

[B18] Jain S. Self W. H. Wunderink R. G. Fakhran S. Balk R. Bramley A. M.. (2015). Community-acquired pneumonia requiring hospitalization among U.S. Adults. N Engl. J. Med. 373, 415–427. doi: 10.1056/NEJMoa1500245 26172429 PMC4728150

[B19] Kalbian I. Park J. W. Goswami K. Lee Y.-K. Parvizi J. Koo K.-H. (2020). Culture-negative periprosthetic joint infection: prevalence, aetiology, evaluation, recommendations, and treatment. Int. Orthopaedics (SICOT). 44, 1255–1261. doi: 10.1007/s00264-020-04627-5 32449042

[B20] Klosterman M. M. Villani M. C. Hamilton E. C. Jo C. Copley L. A. (2021). Primary septic arthritis in children demonstrates presumed and confirmed varieties which require age-specific evaluation and treatment strategies. J. Pediatr. Orthop. 42, e27–e33. doi: 10.1097/bpo.0000000000001976 34560764

[B21] Krauss J. L. Roper P. M. Ballard A. Shih C.-C. Fitzpatrick J. A. J. Cassat J. E.. (2019). Staphylococcus aureus infects osteoclasts and replicates intracellularly. MBio 10, e02447-19. doi: 10.1128/mBio.02447-19 31615966 PMC6794488

[B22] Krauth D. S. Barlow B. T. Berjohn C. M. (2021). Fungal osteomyelitis and septic arthritis in an immune competent man: The first report of invasive osteoarticular infection due to Scedosporium dehoogii. Med. Mycol Case Rep. 33, 14–17. doi: 10.1016/j.mmcr.2021.06.001 34258180 PMC8253999

[B23] Lasso B. M. Pérez G. J. (2009). Pericarditis por Mycobacterium tuberculosis multiresistente en un paciente con infección por VIH: Reporte de un caso clínico y revisión de la literatura. Rev. Chil Infectol 26, 156–161. doi: 10.4067/s0716-10182009000200008 19621148

[B24] Li H. Gao H. Meng H. Wang Q. Li S. Chen H.. (2018). Detection of pulmonary infectious pathogens from lung biopsy tissues by metagenomic next-generation sequencing. Front. Cell Infect. Microbiol. 8, 205. doi: 10.3389/fcimb.2018.00205 29988504 PMC6026637

[B25] Mei J. Hu H. Zhu S. Ding H. Huang Z. Li W.. (2023). Diagnostic role of mNGS in polymicrobial periprosthetic joint infection. JCM. 12, 1838. doi: 10.3390/jcm12051838 36902625 PMC10003677

[B26] Miao Q. Ma Y. Wang Q. Pan J. Zhang Y. Jin W.. (2018). Microbiological diagnostic performance of metagenomic next-generation sequencing when applied to clinical practice. Clin. Infect. Dis. 67, S231–S240. doi: 10.1093/cid/ciy693 30423048

[B27] Mortazavi S. M. J. Vegari D. Ho A. Zmistowski B. Parvizi J. (2011). Two-stage exchange arthroplasty for infected total knee arthroplasty: predictors of failure. Clin. Orthop Relat. Res. 469, 3049–3054. doi: 10.1007/s11999-011-2030-8 21866421 PMC3183212

[B28] Murugan K. Vasudevan N. (2018). Intracellular toxicity exerted by PCBs and role of VBNC bacterial strains in biodegradation. Ecotoxicol Environ. Saf. 157, 40–60. doi: 10.1016/j.ecoenv.2018.03.014 29605643

[B29] Qian M. Li C. Zhang M. Zhan Y. Zhu B. Wang L.. (2024). Blood metagenomics next-generation sequencing has advantages in detecting difficult-to-cultivate pathogens, and mixed infections: results from a real-world cohort. Front. Cell Infect. Microbiol. 13. doi: 10.3389/fcimb.2023.1268281 PMC10768086 38188631

[B30] Reisener M. Perka C. (2018). Do culture-negative periprosthetic joint infections have a worse outcome than culture-positive periprosthetic joint infections? A systematic review and meta-analysis. BioMed. Res. Int. 2018, 1–12. doi: 10.1155/2018/6278012 PMC6077559 30112408

[B31] Rienmüller A. Borens O. (2016). Propionibacterium prosthetic joint infection: experience from a retrospective database analysis. Eur. J. Orthop Surg. Traumatol. 26, 429–434. doi: 10.1007/s00590-016-1766-y 27017334 PMC4856714

[B32] Schwarz E. M. Parvizi J. Gehrke T. Aiyer A. Battenberg A. Brown S. A.. (2019). 2018 International consensus meeting on musculoskeletal infection: research priorities from the general assembly questions. J. Orthop Res. 37, 997–1006. doi: 10.1002/jorr.v37.5 30977537

[B33] Signat B. Roques C. Poulet P. Duffaut D.. (2011). Fusobacterium nucleatum in periodontal health and disease. Curr. Issues Mol. Biol. 13, 25 36. doi: 10.21775/cimb.013.025 21220789

[B34] Singh M. Jeyaraman M. Jeyaraman N. Jayakumar T. Iyengar Karthikeyan P. Jain V. K. (2023). Mycobacterium Tuberculosis infection of the wrist joint: A current concepts review. J. Clin. Orthop Trauma. 44, 102257. doi: 10.1016/j.jcot.2023.102257 37841656 PMC10568419

[B35] Sousa R. Carvalho A. Santos A. C. Abreu M. A. (2021). Optimal microbiological sampling for the diagnosis of osteoarticular infection. EFORT Open Rev. 6, 390–398. doi: 10.1302/2058-5241.6.210011 34267930 PMC8246105

[B36] Street T. L. Sanderson N. D. Atkins B. L. Brent A. J. Cole K. Foster D.. (2017). Molecular diagnosis of orthopedic-device-related infection directly from sonication fluid by metagenomic sequencing. J. Clin. Microbiol. 55, 2334–2347. doi: 10.1128/JCM.00462-17 28490492 PMC5527411

[B37] Suárez-Cuervo C. Nicolás C. Fernández-Suárez J. Morilla A. Fernández J. Caminal-Montero L. (2022). Ureaplasma parvum septic arthritis, a clinic challenge. Diagnostics (Basel). 12, 2416. doi: 10.3390/diagnostics12102416 36292105 PMC9600973

[B38] Tarabichi M. Shohat N. Goswami K. Parvizi J. (2018). Can next generation sequencing play a role in detecting pathogens in synovial fluid? Bone Jt J. 100-B, 127–133. doi: 10.1302/0301-620x.100b2.bjj-2017-0531.r2 29437053

[B39] Thoendel M. Jeraldo P. Greenwood-Quaintance K. E. Chia N. Abdel M. P. Steckelberg J. M.. (2017). A novel prosthetic joint infection pathogen, mycoplasma salivarium, identified by metagenomic shotgun sequencing. Clin. Infect. Dis. 65, 332–335. doi: 10.1093/cid/cix296 28379472 PMC5848249

[B40] Wang J. Han Y. Feng J. (2019). Metagenomic next-generation sequencing for mixed pulmonary infection diagnosis. BMC Pulm Med. 19. doi: 10.1186/s12890-019-1022-4 PMC6921575 31856779

[B41] Wang C. Huang Z. Li W. Fang X. Zhang W. (2020). Can metagenomic next-generation sequencing identify the pathogens responsible for culture-negative prosthetic joint infection? BMC Infect. Dis. 20, 253. doi: 10.1186/s12879-020-04955-2 32228597 PMC7106575

[B42] Watanabe C. Yoshida Y. Kidoguchi G. Kitagawa H. Shoji T. Nakamoto N.. (2023). Disseminated *Mycobacterium abscessus* infection with osteoarticular manifestations as an important differential diagnosis of inflammatory arthritis: A case report and literature review. Mod Rheumatol Case Rep. 8, 49–54. doi: 10.1093/mrcr/rxad054 37718611

[B43] Wolcott R. Costerton J. W. Raoult D. Cutler S. J. (2013). The polymicrobial nature of biofilm infection. Clin. Microbiol. Infect. 19, 107–112. doi: 10.1111/j.1469-0691.2012.04001.x 22925473

[B44] Xiao N. Gai W. Hu W. G. Li J.-X. Zhang Y. Zhao X.-Y. (2019). Next-generation-sequencing technology used for the detection of Mycoplasma hominisin renal cyst fluid: a case report. Infect. Drug Resist. 12, 1073–1079. doi: 10.2147/IDR.S198678 31213854 PMC6537462

[B45] Xie Y. Dai B. Zhou X. Liu H. Wu W. Yu F.. (2023). Diagnostic value of metagenomic next-generation sequencing for multi-pathogenic pneumonia in HIV-infected patients. Infect. Drug Resist. 16, 607–618. doi: 10.2147/IDR.S394265 36733920 PMC9888013

[B46] Xie B. Guo R. Yang X. Wan L. Yao W. Lai Q.. (2020). Epidemiology and drug resistance analysis of mixed infection in orthopedic surgical sites. Surg. Infect. (Larchmt). 21, 465–471. doi: 10.1089/sur.2019.276 31895669 PMC7247030

[B47] Xie G. Zhao B. Wang X. Bao L. Xu Y. Ren X.. (2021). Exploring the clinical utility of metagenomic next-generation sequencing in the diagnosis of pulmonary infection. Infect. Dis. Ther. 10, 1419–1435. doi: 10.1007/s40121-021-00476-w 34117999 PMC8322361

[B48] Xu H. Roberts N. Singleton F. L. Attwell R. W. Grimes D. J. Colwell R. R. (1982). Survival and Viability of Nonculturable Escherichia coli and Vibrio cholerae in the Estuarine and Marine Environment. Microbial Ecology. 8, 313–323. doi: 10.1007/BF02010671 24226049

[B49] Yagupsky P. (2019). Microbiological diagnosis of skeletal system infections in children. CPR. 15, 154–163. doi: 10.2174/1573396315666190408114653 30961502

[B50] Yagupsky P. Ceroni D. (2023). Editorial: An update on pediatric skeletal system infections. Front. Pediatr. 11. doi: 10.3389/fped.2023.1128126 PMC9969156 36861071

[B51] Zappe B. Graf S. Ochsner P. E. Zimmerli W. Sendi P. (2007). Propionibacterium spp. in prosthetic joint infections: a diagnostic challenge. Arch. Orthop Trauma Surg. 128, 1039–1046. doi: 10.1007/s00402-007-0454-0 17874243

[B52] Zhang C. Fang X. Huang Z. Li W. Zhang C. Yang B.. (2019). Value of mNGS in sonication fluid for the diagnosis of periprosthetic joint infection. Arthroplasty 1, 9. doi: 10.1186/s42836-019-0006-4 35240758 PMC8796449

