# Supplementary material for: Shedding light on negative cultures in osteoarticular infections: leveraging mNGS to unravel risk factors and microbial profiles
Source: Front Cell Infect Microbiol. 2024 Nov 25;14:1457639. doi: 10.3389/fcimb.2024.1457639 (PMC11625738; doi:10.3389/fcimb.2024.1457639)
Supplement: Supplementary file 1 [file Table1.docx]

Table S1. Relative abundance and frequency incidence in CN cases. Relative abundance refers to percent composition of each species relative to the total number of organisms detected within each patient sample.

| Pathogens | Mean relative abundance  (%) | Incidence  (Cases) |
| --- | --- | --- |
| Staphylococcus_aureus | 15.47 | 12 |
| Staphylococcus_epidermidis | 12.01 | 12 |
| Pseudomonas_aeruginosa | 9.99 | 10 |
| Pseudomonas_monteilii | 1.61 | 9 |
| Citrobacter_freundii | 1.65 | 8 |
| Mycoplasma_hominis | 73.67 | 4 |
| Streptococcus_agalactiae | 48.05 | 4 |
| Clostridiales_bacterium_VE202_07 | 1.67 | 3 |
| Enterobacter_kobei | 0.90 | 3 |
| Xanthomonas_campestris | 4.70 | 3 |
| Coxiella_burnetii | 2.08 | 3 |
| Cupriavidus nantongensis | 0.22 | 3 |
| Prevotella_salivae | 0.19 | 3 |
| Enterococcus_faecalis | 17.67 | 3 |
| Enterococcus_7L76 | 12.29 | 3 |
| Staphylococcus_haemolyticus | 6.12 | 2 |
| Propionibacterium_humerusii | 8.59 | 2 |
| Staphylococcus_warneri | 1.46 | 2 |
| Acidovorax_JS42 | 1.28 | 2 |
| Malassezia_globosa | 15.21 | 2 |
| Streptococcus_constellatus | 4.73 | 2 |
| Prevotella_bivia | 7.22 | 2 |
| Prevotella_disiens | 1.22 | 2 |
| Streptococcus_intermedius | 1.38 | 2 |
| Dialister_micraerophilus | 1.06 | 2 |
| Peptoniphilus_lacrimalis | 0.93 | 2 |
| solobacterium moorei | 1.00 | 2 |
| Staphylococcus_lugdunensis | 6.09 | 2 |
| Staphylococcus_pasteuri | 1.53 | 2 |
| Klebsiella_pneumoniae | 3.08 | 2 |
| Bacteroides_fragilis | 24.49 | 2 |
| Bacteroides_ovatus | 0.96 | 2 |
| Klebsiella_quasipneumoniae | 0.98 | 2 |
| Streptococcus_dysgalactiae | 19.67 | 2 |
| Burkholderia | 0.44 | 2 |
| Flavobacterium_johnsoniae | 0.60 | 1 |
| Staphylococcus_cohnii | 0.39 | 1 |
| Acinetobacter_bereziniae | 0.24 | 1 |
| Salmonella_enterica | 74.00 | 1 |
| Finegoldia_magna | 0.49 | 1 |
| Clostridium_sticklandii | 0.00 | 1 |
| Agrobacterium_fabrum | 0.88 | 1 |
| Mesorhizobium_australicum | 0.15 | 1 |
| Mesorhizobium_ciceri | 0.07 | 1 |
| Rhizobium_leguminosarum | 0.01 | 1 |
| Enterobacter_xiangfangensis | 8.42 | 1 |
| Pantoea_dispersa | 0.41 | 1 |
| Klebsiella_aerogenes | 0.50 | 1 |
| Klebsiella_oxytoca | 0.51 | 1 |
| Erwinia_mallotivora | 0.30 | 1 |
| Edwardsiella_ictaluri | 0.04 | 1 |
| Lelliottia_ammigena | 1.00 | 1 |
| Corynebacterium_striatum | 1.00 | 1 |
| Penicillium_chrysogenum | 8.30 | 1 |
| Anaerococcus_prevotii | 0.83 | 1 |
| Enterococcus_casseliflavus | 1.08 | 1 |
| Corynebacterium_tuberculostearicum | 0.26 | 1 |
| Porphyromonas_endodontalis | 0.25 | 1 |
| Acinetobacter_gyllenbergii | 2.50 | 1 |
| Varibaculum_cambriense | 1.00 | 1 |
| Streptococcus_pseudopneumoniae | 1.40 | 1 |
| Brevibacterium_epidermidis | 9.28 | 1 |
| Brevibacterium_linens | 2.82 | 1 |
| Raoultella_ornithinolytica | 0.21 | 1 |
| Sphingobium_yanoikuyae | 2.12 | 1 |
| Cupriavidus_pauculus | 0.75 | 1 |
| Mycoplasma_bovis | 0.01 | 1 |
| Staphylococcus_pseudintermedius | 1.68 | 1 |
| Acinetobacter_soli | 0.23 | 1 |
| Pseudomonas_denitrificans | 0.49 | 1 |
| Alternaria_alternata | 14.32 | 1 |
| Meyerozyma_guilliermondii | 21.93 | 1 |
| Malassezia_furfur | 10.13 | 1 |
| Chaetomium_globosum | 2.25 | 1 |
| Neurospora_tetrasperma | 2.69 | 1 |
| Prevotella_buccae | 0.03 | 1 |
| Parabacteroides_distasonis | 0.26 | 1 |
| Parabacteroides_johnsonii | 0.03 | 1 |
| Streptococcus_acidominimus | 0.22 | 1 |
| Facklamia_hominis | 0.01 | 1 |
| Streptococcus_peroris | 0.20 | 1 |
| Streptococcus_urinalis | 7.00 | 1 |
| Burkholderia_cenocepacia | 0.80 | 1 |
| Mycobacterium_colombiense | 13.34 | 1 |
| Staphylococcus_saccharolyticus | 0.20 | 1 |
| Enterobacter_hormaechei | 7.34 | 1 |
| Pseudomonas_TKP | 2.00 | 1 |
| Delftia_Cs1 | 0.57 | 1 |
| Ralstonia_insidiosa | 1.78 | 1 |
| Sphingobium_yanoikuyae | 2.12 | 1 |
| Sordaria_macrospora | 14.64 | 1 |
| Penicillium_citrinum | 1.32 | 1 |
| Atopobium_parvulum | 7.14 | 1 |
| Bifidobacterium_dentium | 1.62 | 1 |
| Escherichia_coli | 1.26 | 1 |
| Mycobacterium_abscessus | 0.01 | 1 |
| Eggerthella_lenta | 0.32 | 1 |
| Mycobacterium_tuberculosis | 0.52 | 1 |
| Streptococcus_pyogenes | 1.37 | 1 |
| Anaerococcus_hydrogenalis | 0.17 | 1 |
| Pseudomonas_entomophila | 0.27 | 1 |
| Candida_parapsilosis | 1.50 | 1 |
| Methylobacterium_populi | 0.09 | 1 |
| Acinetobacter_pittii | 0.96 | 1 |
| Enterobacter_cloacae | 4.92 | 1 |
| Acinetobacter_calcoaceticus | 0.37 | 1 |
| Aeromonas_caviae | 0.87 | 1 |
| Alternaria_alternata | 14.32 | 1 |
| Nosema_bombycis | 9.95 | 1 |
| Albugo_laibachii | 7.80 | 1 |
| Parastagonospora_nodorum | 2.97 | 1 |
| Fusarium_graminearum | 0.63 | 1 |
| Aerococcus_viridans | 0.30 | 1 |
| Moraxella_oblonga | 0.01 | 1 |

Table S2. Representative culture-negative cases.

| Case | Diagnosis | Classification | mNGS | Reason for culture  negative result |
| --- | --- | --- | --- | --- |
| 1 | PJI | IOI | Candida tropicalis | Fastidious bacteria |
| 2 | PJI | IOI | Coxiella burnetii | Fastidious bacteria |
| 3 | PJI | IOI | Fusobacterium nucleatum | Fastidious bacteria |
| 4 | PJI | IOI | Mycoplasma hominis | Fastidious bacteria |
| 5 | PJI | IOI | Finegoldia magna | Fastidious bacteria |
| 6 | PSA | POI | Parvimonas micra | Fastidious bacteria |
| 7 | SA | IOI | Mycobacterium abscessus | Rare pathogens |
| 8 | MTB | POI | Mycobacterium tuberculosis | Rare pathogens |
| 9 | PJI | IOI | Mycobacterium colombiense | Rare pathogens |
| 10 | PJI | IOI | Staphylococcus epidermidis | Prior use of antibiotics |
| 11 | PSA | POI | Staphylococcus aureus | Prior use of antibiotics |
| 12 | PJI | IOI | Staphylococcus aureus | Biofilm formation |
| 13 | PJI | IOI | Pseudomonas aeruginosa | Unknown/VBNC |

mNGS: metagenomic next-generation sequencing; PJI: periprosthetic joint infection; IOI: invasive osteoarticular infection; SA: septic arthritis; MTB: musculoskeletal TB; PSA: Primary septic arthritis; POI: primary osteoarticular infection; VBNC: viable but nonculturable.
